# Attention Deficit Hyperactivity Disorder comorbid oppositional defiant disorder and its predominately inattentive type: evidence for an association with COMT but not MAOA in a Chinese sample

**DOI:** 10.1186/1744-9081-5-8

**Published:** 2009-02-19

**Authors:** Qiu-Jin Qian, Jin Liu, Yu-Feng Wang, Li Yang, Li-Li Guan, Stephen V Faraone

**Affiliations:** 1 Institute of Mental Health, Peking University, Beijing, 10083, China; 2 Key Laboratory of Mental Health, Ministry of Health, Peking University, Beijing, 10083, China; 3 Departments of Psychiatry and Neuroscience & Physiology, SUNY Upstate Medical University, Syracuse, New York, USA

## Abstract

**Background:**

There are three childhood disruptive behavior disorders (DBDs), attention deficit hyperactivity disorder (ADHD), oppositional defiant disorder (ODD), and conduct disorder (CD). The most common comorbid disorder in ADHD is ODD. DSM-IV describes three ADHD subtypes: predominantly inattentive type (ADHD-IA), predominantly hyperactive-impulsive type (ADHD-HI), and combined type (ADHD-C). Prior work suggests that specific candidate genes are associated with specific subtypes of ADHD in China. Our previous association studies between ADHD and functional polymorphisms of COMT and MAOA, consistently showed the low transcriptional activity alleles were preferentially transmitted to ADHD-IA boys. Thus, the goal of the present study is to test the hypothesis that COMT Val158Met and MAOA-uVNTR jointly contribute to the ODD phenotype among Chinese ADHD boys.

**Methods:**

171 Chinese boys between 6 and 17.5 years old (mean = 10.3, SD = 2.6) with complete COMT val158met and MAOA-uVNTR genotyping information were studied. We used logistic regression with genotypes as independent variables and the binary phenotype as the dependent variable. We used p < 0.05 as the level of nominal statistical significance. Bonferroni correction procedures were used to adjust for multiple comparisons.

**Results:**

Our results highlight the potential etiologic role of COMT in the ADHD with comorbid ODD and its predominately inattentive type in male Chinese subjects. ADHD with comorbid ODD was associated with homozygosity of the high-activity Val allele, while the predominantly inattentive ADHD subtype was associated with the low-activity Met allele. We found no evidence of association between the MAOA-uVNTR variant and ADHD with comorbid ODD or the ADHD-IA subtype.

**Conclusion:**

Our study of attention deficit hyperactivity disorder comorbid oppositional defiant disorder and its predominately inattentive type highlights the potential etiologic role of COMT for ADHD children in China. But we failed to observe an interaction between COMT and MAOA, which suggests that epistasis between COMT and MAOA genes does not influence the phenotype of ADHD-IA with comorbid ODD in a clinical sample of Chinese male subjects. To confirm our findings further studies with a larger number of subjects and healthy controls are needed.

## Background

The three childhood disruptive behavior disorders (DBDs), attention deficit hyperactivity disorder (ADHD), oppositional defiant disorder (ODD), and conduct disorder (CD), are characterized by developmentally extreme and impairing levels of distractibility, hyperactivity, impulsivity, hostility, and aggression [1]. They account for a significant number of child psychiatry referrals [2]. The most common comorbid disorder in ADHD is ODD, with our prior work reporting that 36.5% of children with ADHD have ODD in a sample of Chinese outpatients [3].

DSM-IV for ADHD describes three subtypes: predominantly inattentive type (ADHD-IA), predominantly hyperactive-impulsive type (ADHD-HI), and combined type (ADHD-C), each with different characteristics and correlates [1]. Although ADHD is characterized by core symptoms (inattention, hyperactivity and impulsivity), there is considerable heterogeneity in clinical features among individuals with ADHD [4]. Some twin studies have demonstrated that the transmission of inattentive and hyperactive-impulsive symptoms is distinct [5,6]. With a heritability estimate of 0.76 [7], evidence that the etiology of ADHD is partially due to genes is compelling. The evidence that there may be genes associated with specific ADHD subtypes [6,8] supported the heterogeneity inherent in the definition of ADHD. Research has found that specific candidate genes are associated with specific subtypes of ADHD in China. In our prior work, the Met allele of COMT was over transmitted to ADHD boys, especially the ADHD-IA subtype, and the -697G allele and haplotype -759C/-697G of HTR2C was significantly over transmitted to ADHD-C probands [9-11].

Because there is evidence that the covariation between ADHD, ODD, and CD is largely due to shared genetic influences [12,13], it is likely that at least some genes are related to general susceptibility to DBDs [13], and that dopaminergic genes may influence all of the externalizing disorders.

Monoamine neurotransmitters in the brain, including dopamine (DA), noradrenalin (NE), and serotonin (5-HT), are metabolized by two key enzymes, catechol-O-methyltransferase (COMT) and monoamine oxidase (MAO). These two enzymes are of interest in the study of aggressive behavior in both animals and human subjects.

The human COMT gene on 22q11 contains a functional polymorphism (Val158Met) affecting the activity of the enzyme at body temperature [14,15]. The low-activity Met allele results in slower inactivation of extracellular dopamine within the brain, especially in prefrontal cortex [15,16]. There are data showing gender-specific effects of COMT Val158Met genetic polymorphism on measures of cognitive function [17]. We have previously reported the low enzyme activity COMT Met allele was preferentially transmitted to ADHD boys but not girls [9]. Early-onset antisocial behavior in a high risk clinical group is predicted by COMT Val158Met and birth weight, in which children with the ValVal genotype were more susceptible to the effects of lower birth weight, had more symptoms of conduct disorder and more aggressive behaviors compared with Met carriers [18,19], although one study gave negative results [20].

The gene for monoamine oxidase A (MAOA) maps to Xp11.23. The longer repeats (3.5R, 4R, and 5R) of a functional polymorphism in the promoter region of the MAOA gene consists of 30-bp upstream repeats (MAOA-uVNTR). The long alleles have been shown to affect the transcription of the gene three to four times more efficiently than the shorter 3R allele [21,22]. Studies in both humans and animal models have supported the involvement of MAOA in the etiology of externalizing behaviors, especially in males, including impulsivity and aggression [23-26], and ADHD [27]. In our research group, Zhang et al., found a significant preferential transmission of the 3R allele of MAOA-uVNTR to ADHD among boys (*P *= 0.01), and also the inattentive subtype of ADHD in boys (*P *= 0.02). Zhang et al. found no association between MAOA-uVNTR and ADHD among boys comorbid with ODD.

Since our previous association studies between ADHD clinical phenotypes and these two functional polymorphisms consistently showed the low transcriptional activity alleles were preferentially transmitted to the inattentive subtype of ADHD in boys, and because these polymorphisms are all involved in the probable etiology of externalizing behaviors, the goal of the present study is to test the hypothesis that COMT Val158Met and MAOA-uVNTR jointly contribute to the phenotype of ADHD-IA with comorbid oppositional defiant disorder (ODD) in a clinical sample of Chinese male subjects.

## Methods

### Subjects

ADHD cases were recruited from the child psychiatric clinics at Peking University Institute of Mental Health. Those families who gave consent to participate in the study underwent the assessment process. The study was approved by the Ethics Committee of the Health Science Center, Peking University. To be included in the study, children had to meet three criteria: (1) meet Diagnostic and Statistical Manual of Mental Disorders (DSM-IV) criteria for ADHD[1], (2) have a full scale IQ above 85 according to the Wechsler Intelligence Scale for Chinese Children [28], and (3) be from the male Han population. The exclusion criteria were: cases with any evidence of major neurological conditions or a primary diagnosis of schizophrenia, affective disorder, pervasive development disorder, or epilepsy.

### Assessments

Consensus diagnoses were made according to the Clinical Diagnostic Interviewing Scale (CDIS) [29], a structured interview based on the DSM-IV. The CDIS assesses behavioral and emotional disorders during childhood, including ADHD. The CDIS was translated into Chinese by our group. This rating scale has good sensitivity (97.2%) and specificity (100%) for ADHD. The test-retest reliability and criterion validity were each 0.89. The inter-rater reliability kappa coefficient was 0.74 (P < 0.01) [3]. The CDIS assesses the three DSM-IV subtypes of ADHD: ADHD inattentive type (ADHD-IA), ADHD hyperactive-impulsive type (ADHD-HI), and ADHD combined type (ADHD-C). All the parents were interviewed by two psychiatrists separately, including one senior psychiatrist. Teachers completed Rutter's Scale to evaluate the children's behaviors. Finally, consensus diagnoses were assigned to all probands.

The intelligence quotient (IQ) of subjects was assessed using the Chinese-Wechsler Intelligence Scale for Children (C-WISC), a revised edition of the Wechsler Intelligence Scale for Children-Revised (WISC-R), which was standardized by Gong and Cai [28].

### Genotyping

Genomic DNA was extracted from whole venous blood using the protein-depositing method. The COMT val158met (rs4680) was genotyped under conditions previously described [9]. The MAOA-uVNTR was genotyped according to the protocols described previously [21]. Ambiguous or unidentifiable results were reamplified and rescored. Samples that continued to amplify poorly were eliminated from the study. All genotyping was blinded to the subject's ADHD status and IQ score and all clinical assessments were blinded to genotype status.

### Statistics

We used logistic regression with genotypes as independent variables and the binary phenotype as the dependent variable. We used p < 0.05 as the level of nominal statistical significance. Bonferroni correction procedures were used to adjust for multiple comparisons.

## Results

There were 171 Chinese boys between 6 and 17.5 years old (mean = 10.3, SD = 2.6) with complete COMT val158met and MAOA-uVNTR genotyping information. ADHD-IA accounted for 53.2% of the sample, ADHD-C 39.8%, and ADHD-HI 7.0%. Many ADHD children also met diagnostic criteria for other disorders: 32.7% had comorbid ODD, 6.4% had comorbid CD, 13.4% had an emotional disorder, 13.5% had a tic disorder, 10.5% had an affective disorder, and 32.2% had a learning disability (LD).

### Frequency of allele and genotype

For the COMT Val158Met polymorphism, 171 ADHD boys were successfully genotyped. The allele frequencies were: Val allele 66.1%, Met 33.9%. The genotype frequencies were: valval 42.7%, ValMet 46.8%, MetMet 10.5%. For the MAOA-uVNTR, 185 ADHD boys were successfully genotyped. The 1, 2 and 5 R alleles of MAOA-uVNTR were rare. Therefore, four individuals who carried them (one boy had the 1R allele, two boys had the 2R allele, and one boy had the 5R allele) were excluded. Since the MAOA gene is X-linked, males had only one allele. The alleles were grouped as 3R and 4R in the males for statistical analyses. The allele frequencies were: 3R allele 69.6%, 4R 30.4%. The genotype distributions of the COMT and MAOA gene polymorphism were in Hardy-Weinberg equilibrium (*P *> 0.05). The numbers of subjects in each group are showed in table 1.

**Table 1:** The numbers of subjects in each category of ADHD

ADHD subtype	with ODD	without ODD	total
ADHD -IA	20 (22.0%)	71 (78.0%)	91
ADHD -C, adhd-HI	36 (45.0%)	44 (55.0%)	80

total	56	115	171

### Association between COMT and ODD in ADHD Children

Based on the val158met genotypes, subjects were divided into groups: 73 homozygous for the Val allele and 98 carriers of Met allele since the frequency of the metmet genotype was rare. In the comparison of ADHD with and without ODD, the valval genotype was more frequent among children with ADHD and ODD compared with those having ADHD only (x^2 ^= 5.46. p = 0.019). No statistically significant group differences were found for MAOA-uVNTR (Table 2).

**Table 2:** genotype frequency of COMT and MAOA between two phenotypes

		ADHD with ODD (n = 56)	ADHD without ODD (n = 115)	X^2^	*P*
COMT	ValVal	31 (55.4%)	42 (36.5%)		
	ValMet/MetMet	25 (44.6%)	73 (63.5%)	5.461	0.019
MAOA	3R	38 (67.9%)	81 (70.4%)		
	4R	18 (32.1%)	34 (29.6%)	0.118	0.731

### Gene-gene interaction between COMT and MAOA on ADHD phenotype

Logistic regression was used to assess the joint effects of the COMT and MAOA genes on the risk for ODD. Table 3 shows that the effect of COMT Val158Met was significant after partialing out the effects of MAOA-uVNTR (p = 0.021). The valval genotype increased the risk for ODD 2.2 (1/0.465) times more than the valmet/metmet genotypes. The interaction effect was not significant. The effect of COMT was significant when correcting for the two tests of main effects (p = 0.042), but not when including the interaction effect.

**Table 3:** Logistic regression analysis of ADHD with and without ODD subtype

	β	S.E.	Wald Test	*P*	OR
MAOA-uVNTR	0.109	0.357	0.090	0.764	1.113
COMT Val158Met	-0.766	0.331	5.345	0.021	0.465
Interaction	-0.006	0.724	0.000	0.994	0.994

constant	0.875	1.255	---	---	---

Our previous study showed that COMT met allele and MAOA 3R allele were preferentially transmitted to ADHD boys but not girls, especially the ADHD-IA subtype. In this study, binary logistic regression was used to assess the cumulative and interactive effects of the COMT and MAOA genes on the risk for ADHD subtypes. The dependent factor was the ADHD phenotype with 1 = non ADHD-IA subtype and 2 = ADHD-IA subtype, and the covariates were the genotypes of COMT and MAOA. When comparing the ADHD-IA cases with non ADHD-IA cases, Tables 4 shows that the effects of the COMT risk genotype were significant after partialing out the effects of the MAOA genotype (p = 0.025). The valmet/metmet genotype affected the ADHD-IA subtype 2.024 times more profoundly than the valval genotype. There remained a statistically significant difference when it was adjusted with Bonferroni method (p = 0.050).

**Table 4:** Logistic regression analysis of ADHD-I and non ADHD-I subtype

	β	S.E.	Wald Test	*P*	OR
MAOA-uVNTR	0.019	0.339	0.003	0.956	1.019
COMT Val158Met	0.705	0.314	5.037	0.025	2.024
Interaction	-0.902	0.679	1.767	0.184	0.406

constant	-1.015	1.242	---	---	---

## Discussion

Our results highlight the potential etiologic role of COMT in the ADHD comorbid ODD and its predominately inattentive type in male Chinese subjects. ADHD with comorbid ODD was associated with homozygosity of the high-activity Val allele, while the predominantly inattentive ADHD subtype was associated with the low-activity Met allele.

Catechol-*O*-methyltransferase methylates released catecholamines, including dopamine, as part of the catabolic cascade of this neurotransmitter to homovanillic acid. Two main COMT protein isoforms exist, the short, soluble cytoplasmic (S-COMT) isoform predominates in most assayed tissues [30], while a longer membrane bound form (MB-COMT) is the major species in brain [31]. Although expressed widely, COMT appears to be a minor player in dopamine clearance compared with neuronal synaptic uptake by the dopamine transporter and subsequent monoamine oxidase (MAO) metabolism [32]. However, studies in rats, knockout mice, and monkeys suggest that COMT is of particular importance for intrasynaptic dopamine regulation in the prefrontal cortex (PFC) [33,16-36,15], where dopamine transporter expression is low [35]. Mesocortical dopamine inputs to the prefrontal cortex play a critical role in normal cognitive processes, behavior, and motivation [37,38]. There appears to be excessive transmission of the allele that produces more COMT activity and less PFC DA in some psychiatric disorders, which would cause less efficient PFC processing. [39-42]. There were data that indicated an effect of the COMT Val158Met genetic polymorphism on cognitive functions in childhood [43,17], which showed gender-specific effects on measures of IQ and executive function [17]. ADHD children who were homozygous for the valine variant had significantly better sustained attention than those ADHD children possessing at least one copy of the methionine variant [44].

The PFC has also been implicated in the etiology of aggressive and antisocial behavior in children [45]. In the study of Capsi et al. [19], ADHD subjects with the valval genotype had more symptoms of conduct disorder, were more aggressive, and were more likely to be convicted of criminal offenses compared with methionine carriers. Our present study also suggested a role for the Val variant in ADHD boys comorbid with ODD. ODD and CD seemed to be strongly and developmentally related [46]. Studies suggested that the onset of ODD symptoms may be a possible first step toward a life course characterized by the emergence of more serious antisocial and violent acts for some children [47,48]. In a genetic linkage study, Jain et al. [49] found that incorporating ODD and CD into the definition of affected increased evidence for linkage and implicated new chromosomal regions when compared to analyses examining the broader ADHD phenotype.

We found no evidence of association between the MAOA-uVNTR variant and ADHD with comorbid ODD or the ADHD-IA subtype in contrast with previous studies, in which the short variant was found to be associated with impulsivity, aggression, and ADHD with concurrent conduct disorder [50,51].

There were several limitations to this study that bear comment and suggest future directions. First, the major limitation of the study is the small number of individuals with the subtypes of ADHD. Our results, although nominally significant, were not significant after correcting for multiple comparisons. Larger samples, closer to 1000 are needed for more definitive results [52,53]. Second, we focused the study only in male ADHD subjects considering gender differences, as our previous evidence for a sexually dimorphic transmission of COMT Val158Met and MAOA-uVNTR alleles to individuals with ADHD could be a chance finding. Third, we performed the analyses in the ADHD patients without comparing healthy controls, which limits the generalization of these results. Finally, ADHD or related disorders might be accounted for by both genetic and environmental factors such as maternal prenatal smoking or drinking suggested by previous studies [54,55].

## Conclusion

In summary, our study of attention deficit hyperactivity disorder comorbid oppositional defiant disorder and its predominately inattentive type highlights the potential etiologic role of COMT in a Chinese sample. But we failed to observe an interaction between COMT and MAOA, which suggests that epistasis between COMT and MAOA does not influence the phenotype of ADHD-IA with comorbid ODD in a clinical sample of Chinese male subjects. To confirm our findings further studies with a larger number of subjects and healthy controls are needed.

## Competing interests

The authors declare that they have no competing interests.

## Authors' contributions

QJQ and JL contributed equally to this work, they recruited the subjects, made consensus clinical diagnoses, analyzed the data and drafted the manuscript. YFW designed the study and assisted with proof-reading of the manuscript. LY and LLG also recruited the subjects and provided the data necessary for the analysis. SVF revised manuscript critically and assisted with proof-reading of the manuscript. All authors have read and given final approval of the final manuscript.

## References

[B1] American Psychiatric Association (1994). Diagnostic and statistical manual of mental disorders. (DSM-IV).

[B2] Abikoff H, Klein RG (1992). Attention-deficit hyperactivity and conduct disorder: comorbidity and implications for treatment. J Consult Chin Psychol.

[B3] Yang L, Wang YF, Qian QJ, Biederman J, Faraone SV (2004). DSM-IV subtypes of ADHD in a Chinese outpatient sample. J Am Acad Child Adolesc Psychiatry.

[B4] Nigg JT, Willcutt EG, Doyle AE, Sonuga-Barke EJ (2005). Causal Heterogeneity in Attention-Deficit/Hyperactivity Disorder: Do We Need Neuropsychologically Impaired Subtypes?. Biol Psychiatry.

[B5] Todd RD, Rasmussen ER, Neuman RJ, Reich W, Hudziak JJ, Bucholz KK, Madden PA, Heath A (2001). Familiality and heritability of subtypes of attention deficit hyperactivity disorder in a population sample of adolescent female twins. Am J Psychiatry.

[B6] Rasmussen ER, Neuman RJ, Heath AC, Levy F, Hay DA, Todd RD (2004). Familial clustering of latent class and DSM-IV defined attention-deficit/hyperactivity disorder (ADHD) subtypes. J Child Psychol Psychiatry.

[B7] Faraone SV, Perlis RH, Doyle AE, Smoller JW, Goralnick JJ, Holmgren MA, Sklar P (2005). Molecular genetics of attention-deficit/hyperactivity disorder. Biol Psychiatry.

[B8] Neuman R, Todd R, Heath A, Reich W, Hudziak J, Bucholz K, Madden P, Begleiter H, Porjesz B, Kuperman S, Hesselbrock V, Reich T (1999). Evaluation of ADHD typology in three contrasting samples: A latent class approach. J Am Acad Child Adolesc Psychiatry.

[B9] Qian Q, Wang Y, Zhou R, Li J, Wang B, Glatt S, Faraone SV (2003). Family-based and Case-control Association Studies of Catechol-O-Methyltransferase in Attention Deficit Hyperactivity Disorder Suggest Genetic Sexual Dimorphism. Am J Med Genet.

[B10] Li J, Wang Y, Zhou R, Zhang H, Yang L, Wang B, Khan S, Faraone SV (2005). Serotonin 5-HT1B receptor gene and attention deficit hyperactivity disorder in Chinese Han subjects. Am J Med Genet.

[B11] Li J, Wang Y, Zhou R, Zhang H, Yang L, Wang B, Faraone SV (2006). Association between polymorphisms in serotonin 2C receptor gene and attention-deficit/hyperactivity disorder in Han Chinese subjects. Neurosci Lett.

[B12] Nadder TS, Rutter M, Silberg JL, Maes HH, Eaves LJ (2002). Genetic effects on the variation and covariation of attention deficit-hyperactivity disorder (ADHD) and oppositional-defiant disorder/conduct disorder (Odd/CD) symptomatologies across informant and occasion of measurement. Psychol Med.

[B13] Dick DM, Viken RJ, Kaprio J, Pulkkinen L, Rose RJ (2005). Understanding the covariation among childhood externalizing symptoms: Genetic and environmental influences on conduct disorder, attention deficit hyperactivity disorder, and oppositional defiant disorder symptoms. J Abnorm Child Psychol.

[B14] Lachman HM, Papolos DF, Saito T, Yu YM, Szumlanski CL, Weinshilboum RM (1996). Human catechol-O-methyltransferase pharmacogenetics: description of a functional polymorphism and its potential application to neuropsychiatric disorders. Pharmacogenetics.

[B15] Chen J, Lipska BK, Halim N, Ma QD, Matsumoto M, Melhem S, Kolachana BS, Hyde TM, Herman MM, Apud J, Egan MF, Kleinman JE, Weinberger DR (2004). Functional analysis of genetic variation in catechol-O-methyltransferase (COMT): effects on mRNA, protein, and enzyme activity in postmortem human brain. Am J Hum Genet.

[B16] Gogos JA, Morgan M, Luine V, Santha M, Ogawa S, Pfaff D, Karayiorgou M (1998). Catechol-O-methyltransferase-deficient mice exhibit sexually dimorphic changes in catecholamine levels and behavior. Proc Natl Acad Sci USA.

[B17] Barnett JH, Heron J, Ring SM, Golding J, Goldman D, Xu K, Jones PB (2007). Gender-specific effects of the catechol-O-methyltransferase Val108/158Met polymorphism on cognitive function in children. Am J Psychiatry.

[B18] Thapar A, Langley K, Fowler T, Rice F, Turic D, Whittinger N, Aggleton J, Bree M Van den, Owen M, O'Donovan M (2005). Catechol O-methyltransferase gene variant and birth weight predict early-onset antisocial behavior in children with attention-deficit/hyperactivity disorder. Arch Gen Psychiatry.

[B19] Caspi A, Langley K, Milne B, Moffitt TE, O'Donovan M, Owen MJ, Polo Tomas M, Poulton R, Rutter M, Taylor A, Williams B, Thapar A (2008). A replicated molecular genetic basis for subtyping antisocial behavior in children with attention-deficit/hyperactivity disorder. Arch Gen Psychiatry.

[B20] Sengupta SM, Grizenko N, Schmitz N, Schwartz G, Ben Amor L, Bellingham J, de Guzman R, Polotskaia A, Ter Stepanian M, Thakur G, Joober R (2006). COMT Val108/158Met gene variant, birth weight, and conduct disorder in children with ADHD. J Am Acad Child Adolesc Psychiatry.

[B21] Sabol SZ, Hu S, Hamer D (1998). A functional polymorphism in the monoamine oxidase A gene promoter. Hum Genet.

[B22] Deckert J, Catalano M, Syagailo YV, Bosi M, Okladnova O, DiBella D, Noethen MM, Maffei P, Franke P, Fritze J, Maier W, Propping P, Beckmann H, Bellodi L, Lesch KP (1999). Excess of high activity monoamine oxidase A gene promoter alleles in female patients with panic disorder. Hum Mol Genet.

[B23] Cases O, Seif I, Grimsby J, Gaspar P, Chen K, Pournin S, Müller U, Aguet M, Babinet C, Shih JC (1995). Aggressive behavior and altered amounts of brain serotonin and norepinephrine in mice lacking MAOA. Science.

[B24] Saito T, Lachman HM, Diaz L, Hallikainen T, Kauhanen J, Salonen JT, Ryynänen OP, Karvonen MK, Syvälahti E, Pohjalainen T, Hietala J, Tiihonen J (2002). Analysis of monoamine oxidase A (MAOA) promoter polymorphism in Finnish male alcoholics. Psychiatry Res.

[B25] Caspi A, McClay J, Moffitt TE, Mill J, Martin J, Craig IW, Taylor A, Poulton R (2002). Role of genotype in the cycle of violence in maltreated children. Science.

[B26] Huang YY, Cate SP, Battistuzzi C, Oquendo MA, Brent D, Mann JJ (2004). An association between a functional polymorphism in the monoamine oxidase a gene promoter, impulsive traits and early abuse experiences. Neuropsychopharmacology.

[B27] Manor I, Tyano S, Mel E, Eisenberg J, Bachner-Melman R, Kotler M, Ebstein RP (2002). Family-based and association studies of monoamine oxidase A and attention deficit hyperactivity disorder (ADHD): Preferential transmission of the long promoter-region repeat and its association with impaired performance on a continuous performance test (TOVA). Mol Psychiatry.

[B28] Gong YX, Cai TS (1993). Manual of Wechsler Intelligence Scale for Children, Chinese revision (C-WISC).

[B29] Barkley RA (1998). Attention-deficit hyperactivity disorder: A clinical workbook.

[B30] Jeffery DR, Roth JA (1984). Characterization of membrane-bound and soluble catechol-O-methyltransferase from human frontal cortex. J Neurochem.

[B31] Tenhunen J, Salminen M, Lundstrom K, Kiviluoto T, Savolainen R, Ulmanen I (1994). Genomic organization of the human catechol-Omethyltransferase gene and its expression from two distinct promoters. Eur J Biochem.

[B32] Huotari M, Santha M, Lucas LR, Karayiorgou M, Gogos JA, Männistö PT (2002). Effect of dopamine uptake inhibition on brain catecholamine levels and locomotion in catechol-O-methyltransferase-disrupted mice. J Pharmacol Exp Ther.

[B33] Giros B, Jaber M, Jones SR, Wightman RM, Caron MG (1996). Hyperlocomotion and indifference to cocaine and amphetamine in mice lacking the dopamine transporter. Nature.

[B34] Karoum F, Chrapusta SJ, Egan MF (1994). 3-Methoxytyramine is the major metabolite of released dopamine in the rat frontal cortex: reassessment of the effects of antipsychotics on the dynamics of dopamine release and metabolism in the frontal cortex, nucleus accumbens, and striatum by a simple two pool model. J Neurochem.

[B35] Sesack SR, Hawrylak VA, Matus C, Guido MA, Levey AI (1998). Dopamine axon varicosities in the prelimbic division of the rat prefrontal cortex exhibit sparse immunoreactivity for the dopamine transporter. J Neurosci.

[B36] Tunbridge EM, Bannerman DM, Sharp T, Harrison PJ (2004). Catechol-Omethyltransferase inhibition improves set-shifting performance and elevates stimulated dopamine release in the rat prefrontal cortex. J Neurosci.

[B37] Lewis DA, Sesack SR, Levey AI, Rosenberg DR (1998). Dopamine axons in primate prefrontal cortex: specificity of distribution, synaptic targets, and development. Adv Pharmacol.

[B38] Goldman-Rakic PS (1998). The cortical dopamine system: role in memory and cognition. Adv Pharmacol.

[B39] Egan MF, Goldberg TE, Kolachana BS, Callicott JH, Mazzanti CM, Straub RE, Goldman D, Weinberger DR (2001). Effect of COMT Val108/158 Met genotype on frontal lobe function and risk for schizophrenia. Proc Natl Acad Sci USA.

[B40] Weinberger DR, Egan MF, Bertolino A, Callicott JH, Mattay VS, Lipska BK, Berman KF, Goldberg TE (2001). Prefrontal neurons and the genetics of schizophrenia. Biol Psychiatry.

[B41] Goldberg TE, Egan MF, Gscheidle T, Coppola R, Weickert T, Kolachana BS, Goldman D, Weinberger DR (2003). Executive subprocesses in working memory: relationship to catechol-O-methyltransferase Val158Met genotype and schizophrenia. Arch Gen Psychiatry.

[B42] Matsumoto M, Weickert CS, Beltaifa S, Kolachana B, Chen J, Hyde TM, Herman MM, Weinberger DR, Kleinman JE (2003). Catechol-O-methyltransferase COMT. mRNA expression in the dorsolateral prefrontal cortex of patients with schizophrenia. Neuropsychopharmacology.

[B43] Diamond A, Briand L, Fossella J, Gehlbach L (2004). Genetic and neurochemical modulation of prefrontal cognitive functions in children. Am J Psychiatry.

[B44] Bellgrove MA, Domschke K, Hawi Z, Kirley A, Mullins C, Robertson IH, Gill M (2005). The methionine allele of the COMT polymorphism impairs prefrontal cognition in children and adolescents with ADHD. Exp Brain Res.

[B45] Raine A (2002). The role of prefrontal deficits, low autonomic arousal, and early health factors in the development of antisocial and aggressive behavior in children. J Child Psychol Psychiatry.

[B46] Conner DF (2000). Aggression and antisocial behavior in children and adolescents: research and treatment.

[B47] August GJ, Realmuto GM, Joyce T, Hektner JM (1999). Persistence and desistance of oppositional defiant disorder in a community sample of children with ADHD. J Am Acad Child Adolesc Psychiatry.

[B48] Loeber R (1990). Development and risk factors of juvenile antisocial behavior and delinquency. Clinical Psychology Review.

[B49] Jain M, Palacio LG, Castellanos FX, Palacio JD, Pineda D, Restrepo MI, Muñoz JF, Lopera F, Wallis D, Berg K, Bailey-Wilson JE, Arcos-Burgos M, Muenke M (2007). Attention-Deficit/Hyperactivity Disorder and Comorbid Disruptive Behavior Disorders: Evidence of Pleiotropy and New Susceptibility Loci. Biol Psychiatry.

[B50] Manuck SB, Flory JD, Ferrell RE, Mann JJ, Muldoon MF (2000). A regulatory polymorphism of the monoamine oxidase-A gene may be associated with variability in aggression, impulsivity, and central nervous system serotonergic responsivity. Psychiatry Res.

[B51] Lawson DC, Turic D, Langley K, Pay HM, Govan CF, Norton N, Hamshere ML, Owen MJ, O'Donovan MC, Thapar A (2003). Association analysis of monoamine oxidase A and attention deficit hyperactivity disorder. Am J Med Genet.

[B52] Wang WY, Barratt BJ, Clayton DG, Todd JA (2005). Genome-wide association studies: theoretical and practical concerns. Nat Rev Genet.

[B53] Craddock N, Owen MJ, MC O'Donovan (2006). The catechol-O-methyl transferase (COMT) gene as a candidate for psychiatric phenotypes: evidence and lessons. Mol Psychiatry.

[B54] Kahn R, Khoury J, Nichols WC, Lanphnear B (2003). Role of dopamine transporter genotype and maternal prenatal smoking in childhood hyperactive-impulsive, inattentive, and oppositional behaviors. J Pediatrics.

[B55] Brookes KJ, Mill J, Guindalini C, Curran S, Xu X, Knight J, Chen CK, Huang YS, Sethna V, Taylor E, Chen W, Breen G, Asherson P (2006). A common haplotype of the dopamine transporter gene associated with attention-deficit/hyperactivity disorder and interacting with maternal use of alcohol during pregnancy. Arch Gene Psychiatry.

